# Influence of Novel Microcapsulates of Bee Products on Gut Microbiota Modulation and Their Prebiotic and Pro-Adhesive Properties

**DOI:** 10.3390/molecules29122751

**Published:** 2024-06-09

**Authors:** Gabriela Kowalska, Justyna Rosicka-Kaczmarek, Karolina Miśkiewicz, Adriana Nowak, Ilona Motyl, Joanna Oracz, Anna Brzozowska, Aleksandra Grzegorczyk, Zuzanna Świniarska

**Affiliations:** 1 Institute of Food Technology and Analysis, Faculty of Biotechnology and Food Sciences, Lodz University of Technology, Stefanowskiego 2/22 Street, 90-537 Lodz, Poland; karolina.miskiewicz@p.lodz.pl (K.M.); joanna.oracz@p.lodz.pl (J.O.); 215945@edu.p.lodz.pl (A.B.); 225276@edu.p.lodz.pl (A.G.); 225436@edu.p.lodz.pl (Z.Ś.); 2 Department of Environmental Biotechnology, Faculty of Biotechnology and Food Sciences, Lodz University of Technology, Wólczańska 171/173 Street, 90-530 Lodz, Poland; adriana.nowak@p.lodz.pl (A.N.); ilona.motyl@p.lodz.pl (I.M.)

**Keywords:** bee products, biopolymeric microparticles, controlled-release systems, prebiotic potential, pro-adhesive capacity, short-chain fatty acids

## Abstract

With the aim to obtain controlled-release systems and to preserve the antioxidant, immunomodulatory, and prebiotic activity of the bioactive compounds, microencapsulation of both honeydew honey and royal jelly into biopolymeric microparticles based on rye bran heteropolysaccharides (HPS) was successfully performed. Honeydew honey and royal jelly microcapsules were prepared by spray-drying method and were characterized in terms of morphology and biological properties. Due to the resistance of the obtained encapsulates to the acidic pH in the stomach and digestive enzymes, the microcapsules showed prebiotic properties positively influencing both the growth, retardation of the dying phase, and the pro-adhesive properties of probiotic bacteria, i.e., *Bifidobacterium* spp. and lactic acid bacteria. Moreover, as a result of fermentation of the microcapsules of bee products in the lumen of the large intestine, an increased synthesis of short-chain fatty acids, i.e., butyric acid, was found on average by 39.2% in relation to the SCFA concentrations obtained as a result of fermentation of native bee products, thus opening new perspectives for the exploitation of honeydew honey and royal jelly loaded microcapsules for nutraceutical applications.

## 1. Introduction

One of the most important features of probiotic bacteria is their ability to adhere [1]. Adhesion is a multi-step process that allows microorganisms to adhere to surfaces. This property determines the process of colonization of the mucosal surface that lines the digestive system. By adhering to the intestinal wall, probiotic microbiota display a beneficial effect on the health of the host by maintaining the intestinal barrier function and showing an immunomodulatory effect; moreover, the adhesive capacity enables the creation of a barrier against intestinal colonization by pathogenic microorganisms [2]. Prebiotics are food ingredients characterized by the ability of stimulating both the growth and activity of probiotic bacteria present in the digestive system [1,3]; however, effective nutrient delivery to microbiome bacteria is challenging due to their susceptibility to degradation in the harsh conditions of the gastrointestinal tract and the need for targeted delivery systems to ensure their viability and efficacy. The main objectives of controlled release are to reduce the loss of target compounds such as nutrients, antioxidants, vitamins, and minerals during digestion, thereby increasing their efficacy and reducing the frequency of dosing. In addition, the active ingredients are released at a controlled rate over an extended period of time at the target site. This ensures a sustained therapeutic effect, improves patient compliance, and minimises side effects by maintaining consistent levels of active compounds in the body [4,5].

According to the available literature data, bee products may show prebiotic properties. It has been proven that chestnut honey can favorably affect the growth of *Lactobacillus acidophilus* and *Lacticaseibacillus rhamnosus* bacteria [5]. It has been shown that linden honey has a positive effect on the probiotic properties of *L. acidophilus* and *L. rhamnosus*, including self-aggregation and hydrophobicity [6], i.e., features that usually largely determine the adhesive capacity of a given strain. Shamala et al. [7] noted a significant increase in the amount of lactic acid bacteria (LAB) in the intestines of rats as a result of honey feeding [6]. Haddadin et al. [8] showed that royal jelly has a beneficial effect on the growth and activity of L. acidophilus and *Bifidobacterium bifidum*. Due to the content of oligosaccharides such as trehalose, kestose, and raffinose, both honey and royal jelly may possess prebiotic properties, stimulating the growth of LAB and bifidobacteria. In addition, bee products are characterized by the presence of antimicrobial ingredients that can act synergistically with probiotics against certain pathogens, ensuring the balance of the microbiome in the body [9,10]. Bee products as prebiotics have a beneficial effect on the survival of probiotics in the gastrointestinal tract, increase the amount of short-chain fatty acids (SCFAs), and increase resistance to pathogens [8].

LAB and bifidobacteria belong to the natural microbiome of the human gastrointestinal tract. These are Gram-positive, heterofermentative bacteria that use primarily lactose, glucose, galactose, sucrose, and fructose as a carbon source. Extra- and intracellular glycolytic enzymes have been identified in the biomass of probiotic bacteria, which are characterized by the ability to catalyze the hydrolysis or transgalactosylation reaction of α- or β-galactosidic bonds. One of the most important features of probiotic bacteria is their ability to adhere [1]. Adhesion is a multi-stage process that allows microorganisms to adhere to surfaces. This property determines the process of colonization of the mucosal surface lining the digestive system. By adhering to the intestinal walls, the probiotic microbiota exerts a beneficial effect on the host’s health by maintaining intestinal barrier function and immunomodulatory effects. Moreover, adhesive abilities enable the creation of a barrier preventing colonization of the intestines by pathogenic microorganisms [2]. Prebiotics are food ingredients characterized by stimulating both the growth and activity of probiotic bacteria present in the digestive system [1,3,11]. The microbiome plays an important role in maintaining the body’s homeostasis, among others, by synthesizing the so-called postbiotics, which include, among others, SCFAs. The analyses mainly cover butyric acid, propionic acid and acetic acid. The molar ratios of the mentioned acids depend on factors including diet, age, and chronic diseases [8]. SCFAs have a number of health-promoting properties. As a source of additional energy for the body, they stimulate the proliferation and differentiation of enterocytes, which are the absorbing cells of the intestinal villi [12,13]. SCFAs also influence the homeostasis of pro- and anti-inflammatory reactions by inducing proliferation and enhancing the functional activity of regulatory T cells, which is achieved by reducing the activity of the histone deacetylase enzyme [14]. Due to the above-mentioned properties, SCFAs are considered an important factor that can support the therapy of chronic inflammatory diseases and in the treatment of intestinal dysbiosis resulting from antibiotic therapy [15]. In addition, SCFAs can demonstrate anti-cancer properties [8,11,16]. SCFAs stimulate the development of intestinal epithelium, hepatocytes, and peripheral tissues, and also contribute to lowering the pH of intestinal contents. It is believed that a low SCFA concentration and high pH of intestinal content predispose to the development of cancer [12]. It has been proven that butyric acid can inhibit the proliferation of cancer cells, induce their apoptosis, and control their cell cycle. Therefore, butyric acid may be an important factor in preventing uncontrolled cell division and, consequently, in the prevention of colorectal cancer [17].

In view of the above, the aim of this work was to obtain—in a spray-drying process—fixed honey and royal jelly preparations in the form of encapsulates with increased bioactive and biological value. Therefore, in order to fully exploit the significant health-promoting potential—including the prebiotic capability—of bee products such as honey and the currently less-appreciated royal jelly, an encapsulation process has been used to obtain systems for the controlled release of the bioactive compounds contained in these products, allowing them to retain their full biological activity. Bioactive heteropolysaccharides (HPS) that have not been used before in the encapsulation process were used as a carrier, i.e., water-soluble arabinoxylans isolated from a by-product produced in the rye milling process—bran. At the same time, the encapsulation process was optimized to obtain microcapsules with the smallest possible ratio of polysaccharide carrier to core. The obtained encapsulates were analyzed for their prebiotic and pro-adhesive properties, which are crucial for the proper formation and functioning of the gastrointestinal microbiome.

## 2. Results and Discussion

### 2.1. Prebiotic Properties of Bee Products and Encapsulates

**Figure 1:**
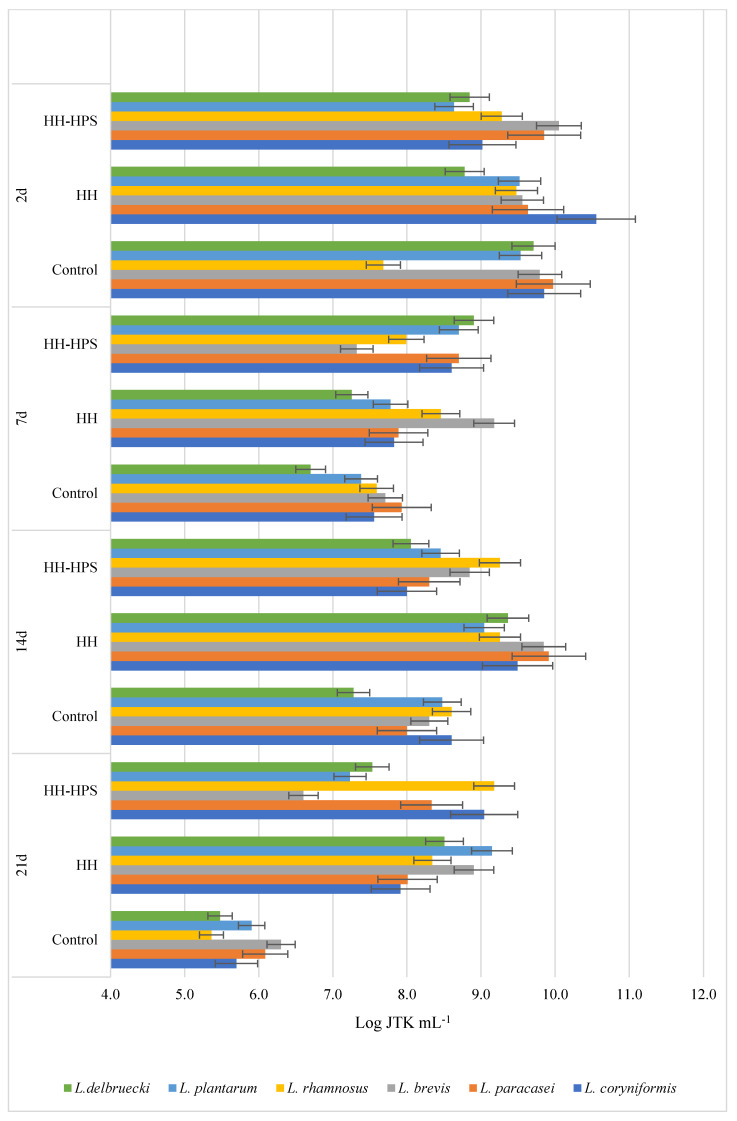
Growth performance of six lactic acid bacteria spp. (abbreviated as L.) in the presence of honeydew honey and its encapsulates depending on the incubation time.

**Figure 2:**
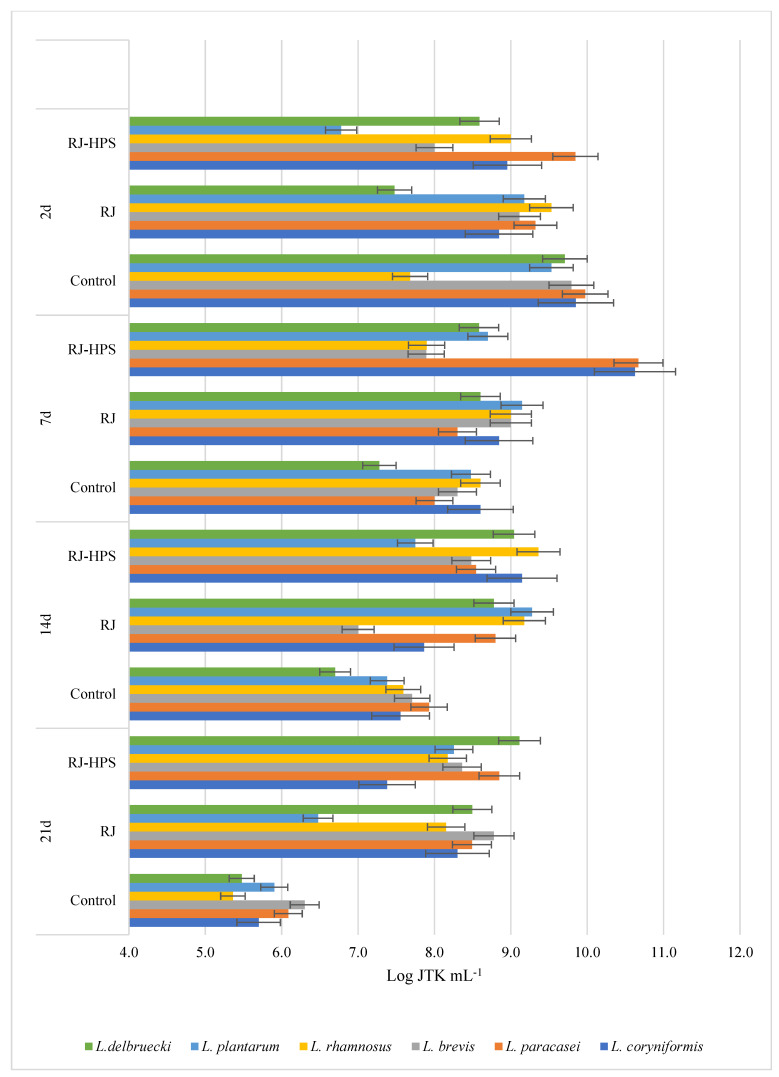
Growth performance of six lactic acid bacteria spp. (abbreviated as L.) in the presence of royal jelly and its encapsulates depending on the incubation time.

**Figure 3:**
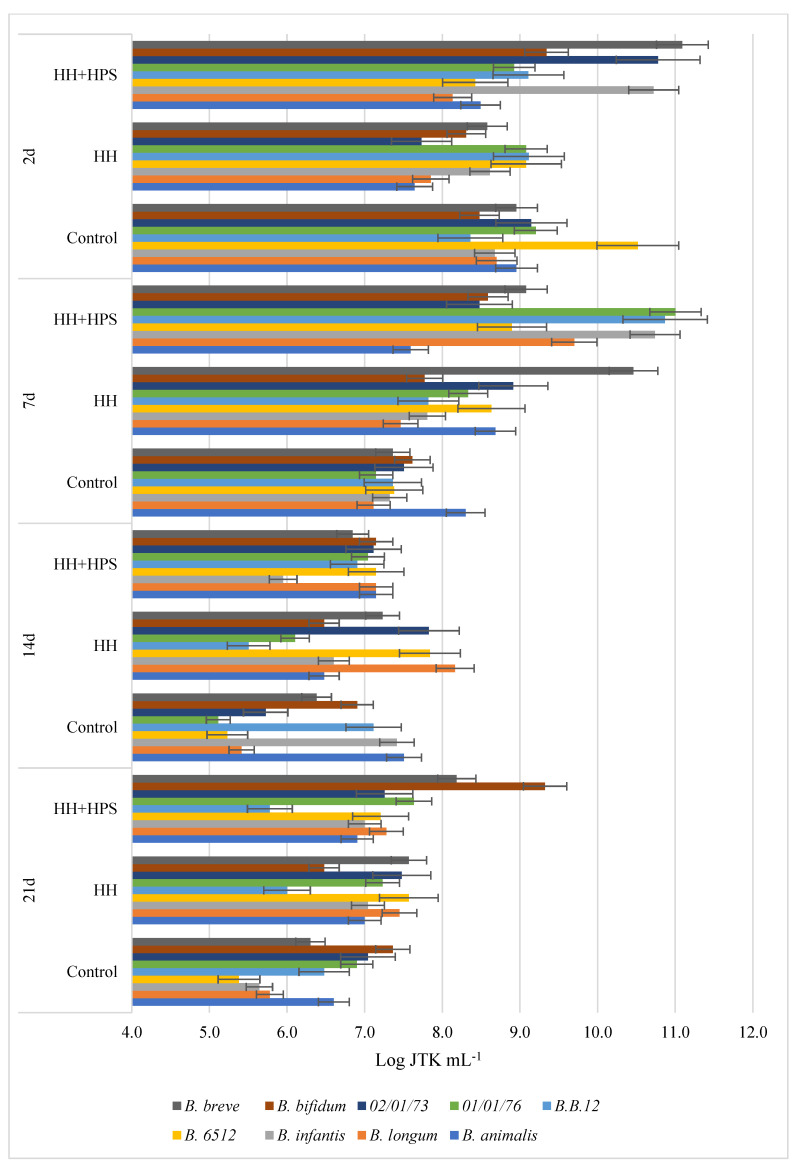
Growth performance of nine *Bifidobacterium* spp. (abbreviated as B.) in the presence of honeydew honey and its encapsulates depending on the incubation time.

**Figure 4:**
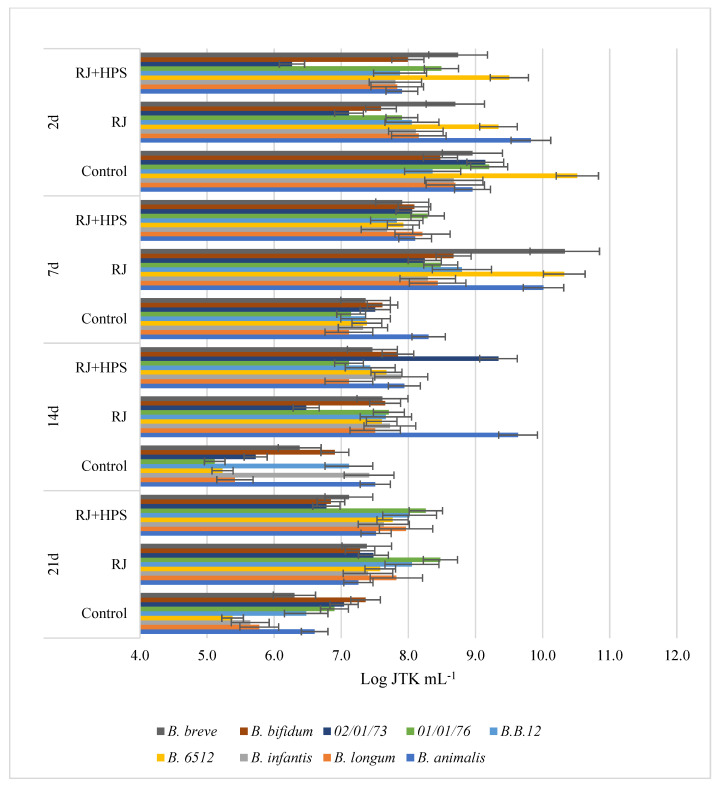
Growth performance of nine *Bifidobacterium* spp. (abbreviated as B.) in the presence of royal jelly and its encapsulates depending on the incubation time.

The results presented in Figure 1, Figure 2, Figure 3 and Figure 4 indicate the effect of honeydew honey, royal jelly, and their microcapsules on the yield of LAB cells and bifidobacteria. It is worth noting that the degree of use of the tested preparations as a carbon source by LAB is a specific feature of a given strain, while changes in the growth rate of *Bifidobacterium* sp. strains are characteristic mainly for individual preparations and depend on the incubation time. Apart from the *L. delbruecki* 0987 strain—for which a reduction in the number of bacteria was observed by 9.3% (both HH- and RJ-HPS)—and the *L. rhamnosus* 0908 strain—for which an increase in the number of bacteria by 23.4% (HH) and 21% (HH-HPS) was observed—in first 2 days of the experiment no significant differences in the growth of LAB bacteria were found depending on the carbon source compared to the control test. In contrast, the growth of *Bifidobacterium* strains in first 2 days of analysis was more diverse. Both honey and its encapsulate HH-HPS showed an average of 16.8% inhibition of the growth of *B.6512* and 8.1% of *B. longum* in 2 days of analysis in relation to the control sample. For the same time of the experiment, an acceleration of the death rate of *Bifidobacterium* spp. (except for *B. animalis* and *B. breve*) in the presence of RJ and RJ-HPS was found compared to the control sample. A significant increase in the number of bacteria was observed on the 7th day of incubation compared to the control sample (bacteria in the MRS) in case of *L. delbruecki* 0987 (HH 9.0%; HH-HPS 32.8%; RJ and RJ-HPS 17.8%), *L. plantarum* 0981 (HH-HPS 17.6% and RJ 7.1%), *L. paracasei* 0993 (HH-HPS 1 0.1% and RJ-HPS 33.8%), and *L. coryniformis* 10AN (HH-HPS 13.2%; RJ-HPS 23.3%). HH-HPS and RJ-HPS also showed a stimulatory effect on the growth of *B. breve* (23.0 and 20.3%, respectively) *B. bifidum* (17.1 and 6.6%), *B.02/01/73* (13.3 and 8.0%), *B.01/01/76* (54.9 and 16.9%), *B.B.12* (47.3 and 18.9%), *B. infantis* (46.6 and 8.2%), and *B. longum* (36.6 and 15.5%) compared to the control (bacteria in *Bifidobacterium* medium).

A particularly significant effect of honeydew honey preparations on the growth of LAB was observed on the 14th and 21st day of incubation. At this time, the protective effects of honey, royal jelly, and their microcapsules are clearly visible, which slow down the rate of LAB and *Bifidobacterium* spp. dieback by an average of 12.6 and 16.2% (14 d) and 46.5 and 16.1% (21 d), respectively, compared to the control sample—in which the dieback phase was observed on the 7th day of the experiment. RJ-HPS (49.9%) had a clear effect slowing down the rate of dying of all strains on the 21st day of the analysis; however, on the 14th day of the experiment, it was observed that native honeydew honey contributed to the inhibition of growth of *B. animalis* and *B.B.12* bacteria by 13.3% and 22.5%. The obtained results are consistent with Carvalho de Melo et al. [18,19], who observed the stimulating effect of four types of monofloral honeys of stingless bees against *L. acidophilus* and *Bifidobacterium* strains. Das et al. [20] showed the prebiotic properties of five Sesamum indicum honeys against *L. acidophilus* [1] bacteria. The results of the research presented by Shamal et al. [7] prove that honey has a stimulating effect on the logarithmic growth rate of *L. acidophilus* and *L. plantarum*.

### 2.2. Pro-Adhesive Potential of Bee Products and Encapsulates

#### 2.2.1. Adhesion to Polystyrene

**Figure 5:**
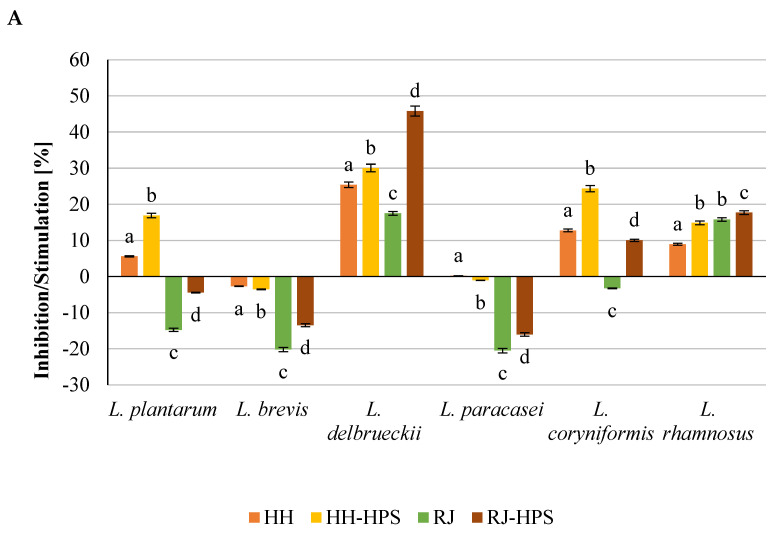

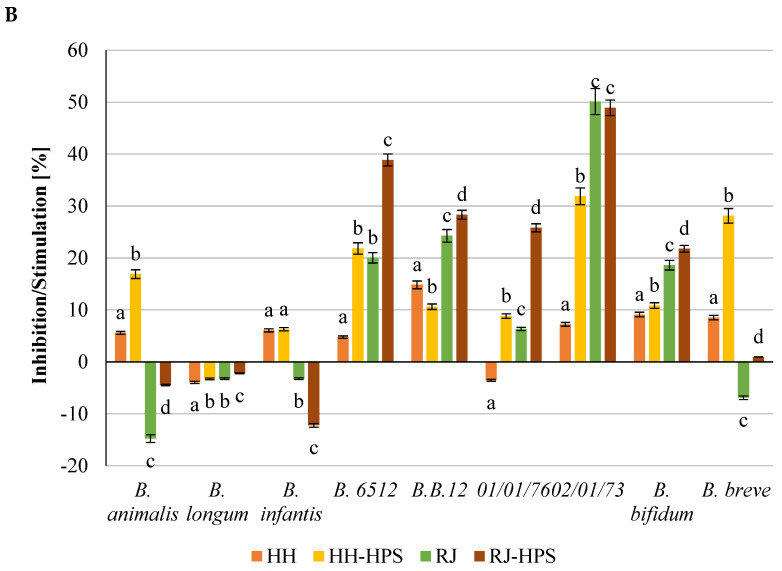
Adherence of (**A**) Lactic acid bacteria and (**B**) Bifidobacterium strains to abiotic polystyrene surface in the presence of honeydew honey, HH-HPS, royal jelly, and RJ-HPS. Data represent means from three to eight repeats (±SD). Results are significantly different for a given strain (ANOVA, *p* < 0.05). B-*Bifidobacterium*.

Polystyrene is one of the most commonly chosen surfaces for testing bacterial adherence [1,2,21]. Both native honey and royal jelly, as well as their encapsulates, stimulated the adhesion of probiotic strains to the polystyrene surface (Figure 5). The influence of the encapsulation of honey and royal jelly on the pro-adhesive properties was dependent on both the strain and the applied core material. The HH-HPS showed a particularly stimulating effect, for which the adhesion stimulation was on average 165.7% for bifidobacteria and 94.5% for LAB strains (excluding *L. brevis* and *L. paracasei* for which an anti-adhesive effect was observed), compared to the statistically significantly lower adhesion of *Bifidobacterium* spp. (5.4%) and LAB (8.4%) recorded in the presence of honey. The HH-HPS stimulated the adhesion of most strains of the genus *Bifidobacterium* (except for *B. infantis*), while RJ-HPS showed a more favorable effect on LAB (117.6% compared to the native royal jelly) adhesion.

Among the preparations, the highest adhesion to polystyrene for *Bifidobacterium* strains was observed in the presence of the HH-HPS, which showed over 3.8-times higher stimulation of the adhesive capacity of *B.6512*, *B.01/01/76*, and *B.02/01/73* than native honeydew honey. The highest adhesion to polystyrene in case of LAB was noticed for *L. coryniformis* 10AN in the presence of RJ-HPS, for which the adhesion stimulation was 407.1% higher than for native royal jelly. Royal jelly encapsulation with HPS biopolymers also intensified the pro-adhesive properties of the core material against *Bifidobacterium* spp., increasing their adhesion by an average of 41.3% compared to bacterial adhesion in the presence of native royal jelly.

#### 2.2.2. Adhesion to Collagen

**Figure 6:**
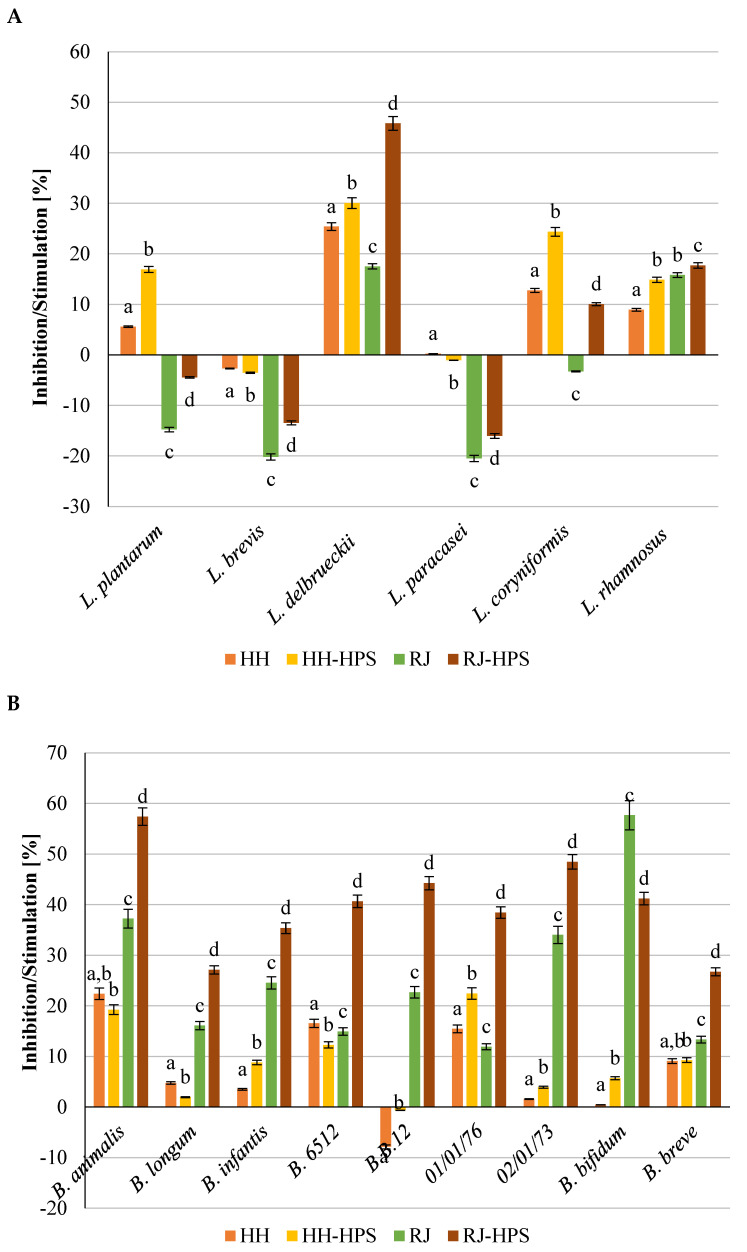
Adherence of (**A**) Lactic acid bacteria and (**B**) Bifidobacterium strains to biotic collagen surface in the presence of honeydew honey, HH-HPS, royal jelly and RJ-HPS. Data represent means from three to eight repeats (±SD). Results are significantly different for a given strain (ANOVA, *p* < 0.05). L-*Lactobacillus*.

The analysis of the adhesion of probiotic bacterial strains to the collagen surface showed a beneficial effect of the encapsulation process on the pro-adhesive properties of the bee products. Apart from honeydew honey and the HH-HPS preparation—which reduced the adhesion of the B.B.12 strain to collagen by 7.9% and 0.7%, respectively, compared to the control sample—bee products and their encapsulates stimulated the degree of adhesion of the tested bifidobacteria strain, as shown in Figure 6B. In addition, all LAB strains (Figure 6A)— *L. paracasei* 0993, *L. coryniformis* 10AN, *L. plantarum* 0981, *L. brevis* 0983, *L. delbrueckii* 0987, and *L. rhamnosus* 0908—showed stronger adhesion to collagen in the presence of HH and HH-HPS compared to the control sample; this is in contrast to RJ and RJ-HPS, which showed an inhibiting effect (on average −12.2%) on all of the LAB strains compared with the control. The effect of bee products and microcapsules on the inhibition/stimulation of bacteria was dependent on the analyzed strain. Among the analyzed samples, the highest stimulation of bacterial adhesion to collagen was found for the RJ-HPS preparation, which increased the adhesion of bifidobacteria by 40.0%, respectively, while native royal jelly caused an average adhesion stimulation of 25.8% for *Bifidobacterium* spp. A particularly significant increase in adhesion resulting from the presence of the RJ-HPS preparation was observed for strains *B. animalis* (57.4%) and *B.02/01/73* (48.5%), for comparison, royal jelly stimulated the adhesion of these strains by 37.2 and 34.0, respectively. Microencapsulation of honey positively impacted the adhesion of both bifidobacteria and LAB strains. Honey encapsulation stimulated the adhesion of *Bifidobacterium* and LAB spp. by 163.7% and 287.4%, respectively. No significant difference in pro-adhesive properties of the RJ-HPS preparation and royal jelly against LAB was observed. Encapsulated royal jelly stimulated the adhesion properties of *Bifidobacterium* spp. in average by 85.7% when comparing with native royal jelly.

#### 2.2.3. Adhesion to mucus from porcine stomach

**Figure 7:**
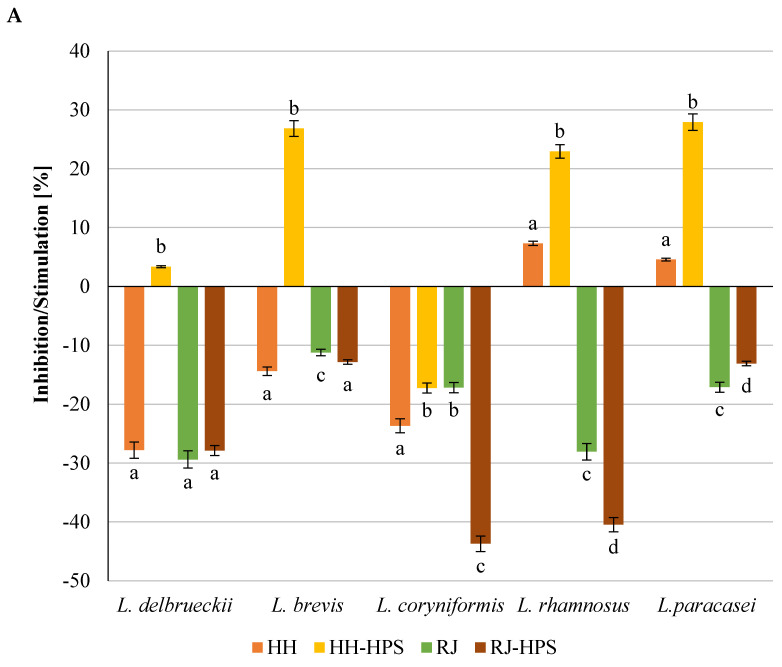

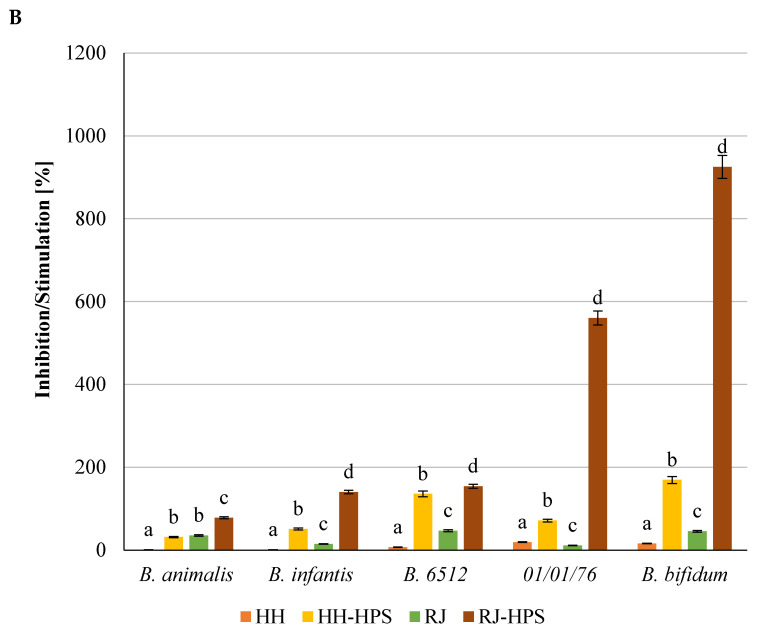
Adherence of (**A**) lactic acid bacteria and (**B**) Bifidobacterium strains to mucus from porcine stomach surface in the presence of honeydew honey, HH-HPS, royal jelly, and RJ-HPS. Data represent means from three to eight repeats (±SD). Results are significantly different for a given strain (ANOVA, *p* < 0.05). B-*Bifidobacterium*.

**Figure 8:**
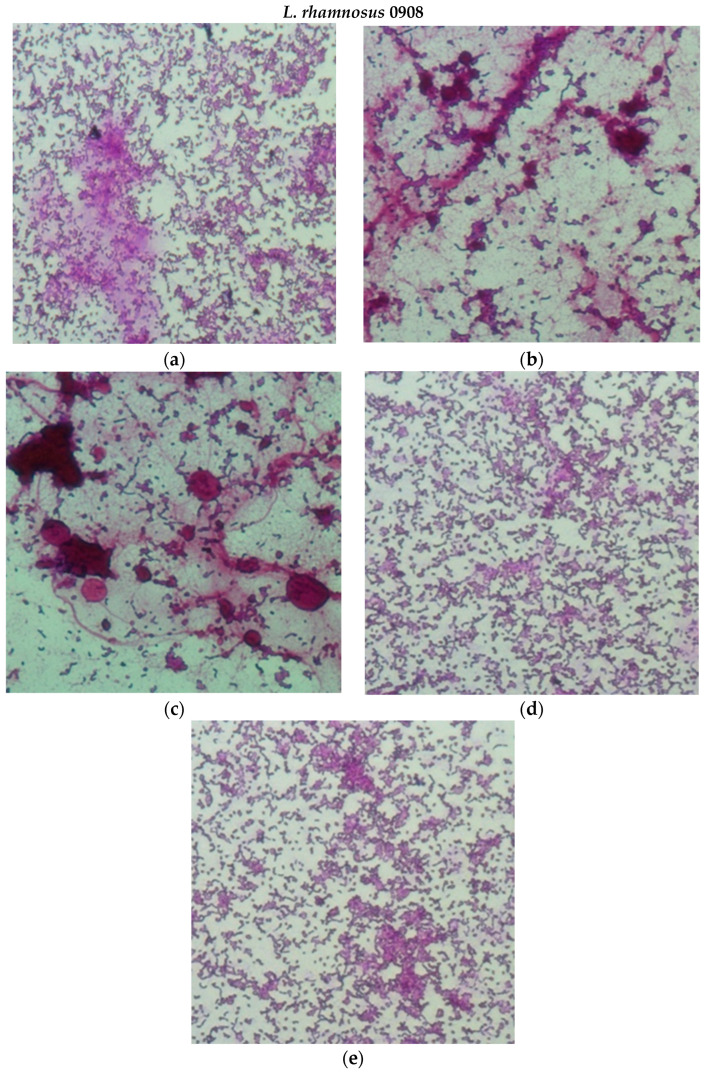

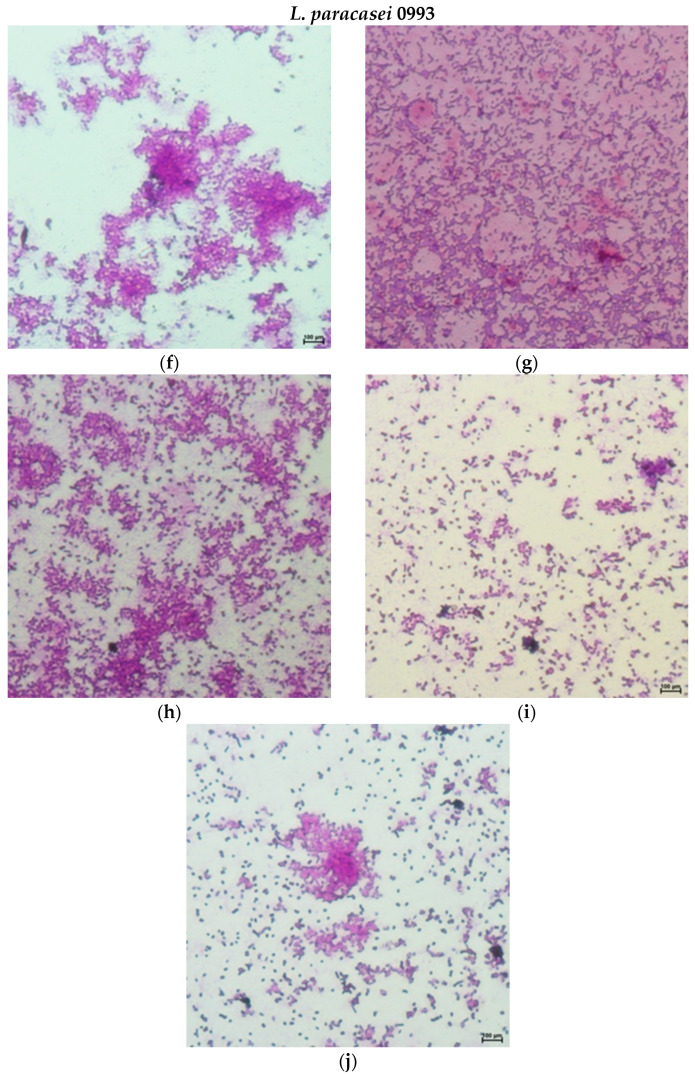
Adherence of *L. rhamnosus* 0908 (**a**–**e**) i *L. paracasei* 0993 (**f**–**j**) to porcine mucous surface. A/F PBS and strain (control), B/G honeydew honey, C/H HH-HPS, D/I royal jelly, and E/J RJ-HPS. Representative microphotographs after staining with 0.1% crystal violet.

The analysis of the adhesion of probiotic bacterial strains to intestinal mucus, simulating the conditions in the human digestive system, showed a beneficial effect of the encapsulation process on the pro-adhesive properties of bee products (Figure 7A,B). Both native bee products and their microcapsules reduced the degree of LAB adhesion (Figure 7A) and simultaneously stimulated the adhesion of all strains of the Bifidobacterium genus (Figure 7B). The HH-HPS preparation stimulated the adhesion of nine out of ten tested strains, including four out of five LAB strains (Figure 7A), while honeydew honey stimulated the adhesion of five out of ten analyzed strains. The average increase in the degree of adhesion in the presence of the HH-HPS preparation was 91.8% (*Bifidobacterium* sp.) and 12.8% (LAB strains); for comparison, the presence of native honeydew honey resulted in adhesion stimulation amounting to an average of 8.8% (*Bifidobacterium* spp.) and inhibition of the degree of adhesion reaching an average of 10.8% (LAB strains) compared to the control. The highest increase in bacterial adhesion in the presence of HH-HPS was recorded for *Bifidobacterium* strains: *B.6512* (135.7%) and *B. bifidum* (169.4%). In the case of LAB, the highest adhesion stimulation was observed for the strains *L. paracasei* 0993 (27.9%) and *L. brevis* 0983 (26.8%). Encapsulation of royal jelly led to a significant increase in the degree of adhesion, especially of strains *B.01/01/76* (560.5%) and *B. bifidum* (925.1%). The mentioned strains in the presence of native royal jelly adhered 11.4% and 45.4% stronger, respectively, than the bacteria in the control sample. Both native royal jelly and RJ-HPS reduced the degree of LAB adhesion by an average of 20.6% and 27.6%. The highest reduction in the degree of adhesion was observed for the strains *L. coryniformis* 10AN (43.7%) and *L. rhamnosus* 0908 (40.5%) in the presence of the RJ-HPS preparation. Microscopic photos showing the adhesion of LAB strains (*L. rhamnosus* 0908 and *L. paracasei* 0993) to intestinal mucus in the presence of preparations (honey **B**, HH-HPS **C**, royal jelly **D**, RJ-HPS **E**) and the control **A** (PBS + strain) are shown in Figure 8.

Based on the results, it can be concluded that the encapsulation process of bee products had a positive effect on the pro-adhesive properties of honey and royal jelly. Microcapsules encapsulated with heteropolysaccharide biopolymers demonstrated particularly favorable pro-adhesive properties towards probiotic bacteria. The analyzed fixed honey and royal jelly preparations stimulated the degree of adhesion of the tested strains more intensively than native bee products.

There are few studies available in the literature discussing the pro-adhesive properties of bee products. Saran et al. [22] assessed the potential prebiotic effect of honey and inulin against two strains of *L. acidophilus* (NCDC 13 and NCDC 291). It was shown that inulin stimulates the autoaggregation ability of the NCDC 13 strain better than NCDC 291, while the *L. acidophilus* NCDC 291 strain showed greater autoaggregation in the presence of honey than *L. acidophilus* NCDC 13. Fratianni et al. [23] analyzed the impact of multifloral honey on inhibition of adhesion abilities and the process of biofilm formation by pathogenic strains of *Acinetobacter baumannii*, *Escherichia coli*, *Listeria monocytogenes*, *Pseudomonas aeruginosa,* and *Staphylococcus aureus* subsp. *aureus* Rosebach. The authors found inhibition of adhesion (up to 84.3%) and inhibition of biofilm formation (up to 93.3%) by pathogens.

### 2.3. Short-Chain Fatty Acids (SCFAs)

Honey, royal jelly, and encapsulates HH-HPS and RJ-HPS were fermented with *Bifidobacterium* and LAB strains. After 48 h, the post-fermentation liquids (metabolites) were qualitatively and quantitatively analyzed for the presence of selected SCFAs, i.e., acetic, propionic, and butyric acids, using the high-performance liquid chromatography (HPLC) method.

**Figure 9:**
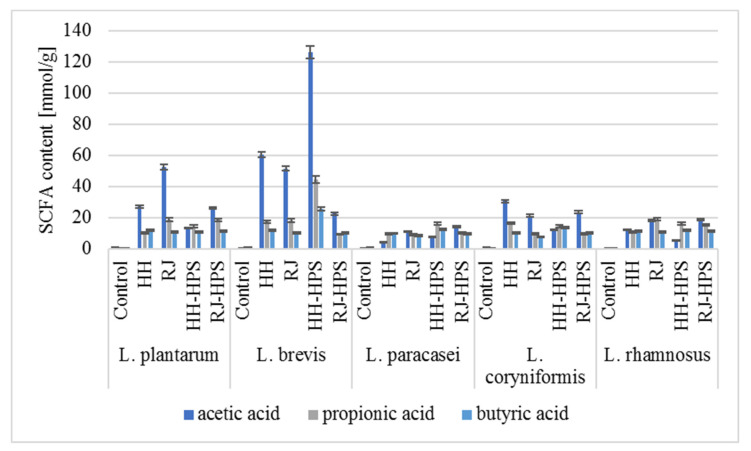
Synthesis of short-chain fatty acids by LAB after 48 h of culture in the presence of honey, royal jelly, bee-product encapsulates. The values shown in the figure are the average of three independent measurements. The size of the standard deviations did not exceed 3% of the obtained values, taking this fact into account, the obtained values are statistically significantly different (*p* ≤ 0.05).

**Figure 10:**
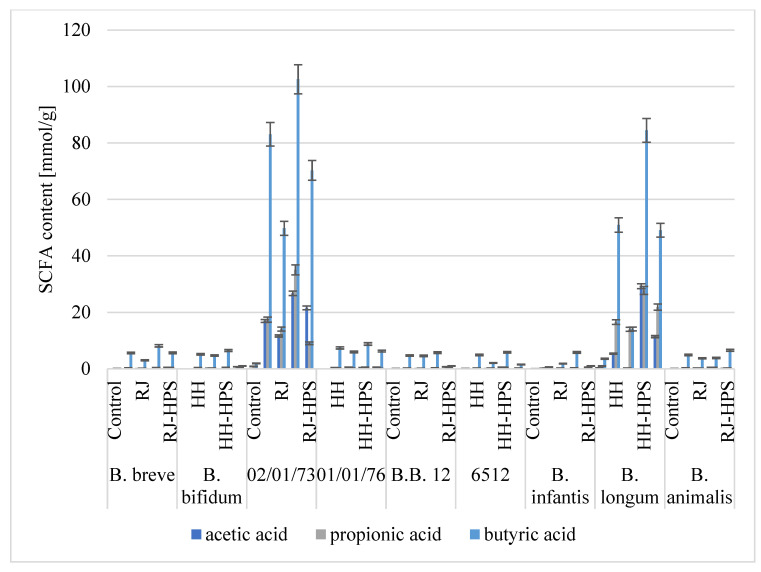
Synthesis of short-chain fatty acids by bifidobacteria after 48 h of culture in the presence of honey, royal jelly, bee-product encapsulates. The values shown in the figure are the average of three independent measurements. The size of the standard deviations did not exceed 3% of the obtained values, taking this fact into account, the obtained values are statistically significantly different (*p* ≤ 0.05).

Based on the HPLC analyses, it can be concluded that both bee products and their encapsulates displayed a beneficial effect on the synthesis of SCFAs (Figure 9 and Figure 10).

Fermentation of honey, royal jelly, and their encapsulates by *L. coryniformis* resulted in a significantly higher SCFA concentration (average 21.17, 12.03, and 11.15 mmol g^−1^ for acetic, propionic, and butyric acids, respectively) than observed for the control sample (0.08, 1.02, and 0.36 mmol g^−1^, respectively). The highest total SCFA concentration was determined after fermentation of HH-HPS and RJ-HPS, for which it was 122.50 and 113.35 mmol g^−1^, respectively. All encapsulates stimulated the synthesis of butyric acid, increasing its concentration by an average of 39.2% compared to the native bee product. The encapsulation process contributed to a reduction in the acetic acid content (by 14.0% on average) but did not significantly change the concentration of propionic acid in honey and royal jelly. The encapsulation process of honey and royal jelly had a positive effect on the synthesis of SCFAs by *L. paracasei* 0993, increasing the concentrations of acetic, propionic, and butyric acids by, on average, 56.4, 34.9, and 19.2% compared to non-encapsulated honey or royal jelly. The encapsulation process contributed 56.4, 34.9, and 19.2% higher content of acetic acid, propionic acid, and butyric acid, respectively, obtained as a result of the metabolism of encapsulates, compared to SCFAs determined as a result of fermentation of the native bee product. After fermentation of honey preparations in the presence of *L. brevis* 0983, a 118.6% higher total SCFA concentration was observed (196.62 mmol g^−1^) compared to the values obtained after fermentation of native honeydew honey (89.98 mmol g^−1^). As a result of fermentation of honey and royal jelly encapsulates by *L. rhamnosus* 0908 and *L. plantarum* 0995, a lower concentration of propionic acid was found (on average by 29.6% and 12.6%) compared to the values determined for native honey and royal jelly; however, the total concentration of SCFAs synthesized as a result of the metabolism of bee-product encapsulates by *L. rhamnosus* 0908 was on average 7.5% higher than that obtained for honey and royal jelly samples. Fermentation of HH-HPS preparations with *L. plantarum* 0995 resulted in 43.1% higher concentrations of propionic acid, respectively, in relation to the values determined after the fermentation process of honeydew honey.

The encapsulation process of honey and royal jelly showed a beneficial effect on the profile of postbiotics obtained as a result of fermentation of fixed bee products by *Bifidobacterium* spp., increasing the total SCFA concentrations by, on average, 13.0–255.7% compared to the total SCFA concentrations recorded as a result of fermentation of native bee products (Figure 10). Fermentation of honey and royal jelly microcapsules with *B. longum*, *B. breve,* and *B. infantis* strains led to a higher total amount of SCFAs on average by 251.3, 114.8, and 255.7%, respectively, compared to the concentrations obtained by fermentation of native bee products. The highest mean total SCFA concentrations recorded from the fermentation of fixed bee products by *B. longum* and *B.02/01/73* strains were 121.78 and 119.26 mmol g^−1^, respectively, compared to that recorded from the fermentation of native honey and royal jelly (amounting to 72.93 and 28.42 mmol g^−1^ for *B. longum* and 117.50 and 75.52 mmol g^−1^ for strain *B.02/01/73*, respectively).

Fermentation of honey, royal jelly, and their encapsulates by *Bifidobacterium* spp. resulted in higher total SCFA concentrations (on average 5.19, 105.97, and 7.26 mmol g^−1^ for *B. infantis*, *B. longum,* and *B. breve*, respectively) than the concentrations determined for the control sample (0.07, 5.08, and 0.21 mmol g^−1^, respectively). The highest total SCFA concentration was determined as a result of fermentation of the HH-HPS (164.30 mmol g^−1^; *B.02/01/73*). This value is 39.8% higher than the concentrations obtained for the fermentation of non-encapsulated core material. The dominant SCFA in this preparation turned out to be butyric acid, the amount of which was 102.54 mmol g^−1^. Such a high total SCFA concentration is the result of the presence of biopolymers, which constitute a good carbon source for SCFA synthesis. A constant molar ratio of acetate, propionate, and butyrate synthesized by LAB as a result of the metabolism of bee products and their encapsulates is noticeable. The concentrations of acetic, propionic, and butyric acids follow the formula proposed by Topping et al. [24]: acetate > propionate = butyrate. It is worth noting that the fermentation process conducted by *Bifidobacterium* spp. also results in a constant that is different from the pattern described above—acetate = propionate < butyrate—and allows us to conclude that the SCFA molar ratio depends not only on diet and chronic diseases but is a specific/strain feature.

According to the available literature data, consumption of royal jelly may promote the growth of *L. acidophilus* and *B. bifidum* bacteria in the colon, and thus lead to the production of larger amounts of SCFAs such as butyric acid, which has been shown in in vitro studies to have anti-inflammatory effects [25]. Upadhyay and Moudgal [26] indicate the potential use of prebiotics in the treatment of acute diarrhea, prevention of post-antibiotic diarrhea, and inflammation of the intestinal mucosa (pouchitis). Haddadin et al. [8] analyzed the effect of three types of mountain multifloral honey on the concentrations of SCFAs synthesized by *B. infantis* and *L. acidophilus*. The dominant acid obtained as a result of the honey fermentation process for all analyzed samples was acetic acid (average 9.8 g L^−1^), followed by propionic acid (2.3 g L^−1^) and butyric acid (1.4 g L^−1^). A constant proportion of acids was noticeable for both *B. infantis* and *L. acidophilus*. Mohan et al. [27] carried out the process of anaerobic lactic fermentation of manuka honey using the *Limosilactobacillus reuteri* strain. Using NMR analysis, the authors also noted the presence of acetic acid (60 mM), succinic acid (20 mM), and propionic acid (<10 mM). There are no reports in the scientific literature regarding the influence of honey or royal jelly microcapsules on the synthesis of SCFAs by probiotic strains.

### 2.4. Surface Morphology Assessment with Scanning Electron Microscopy (SEM)

#### Assessment of the surface morphology of microcapsules

**Figure 11:**
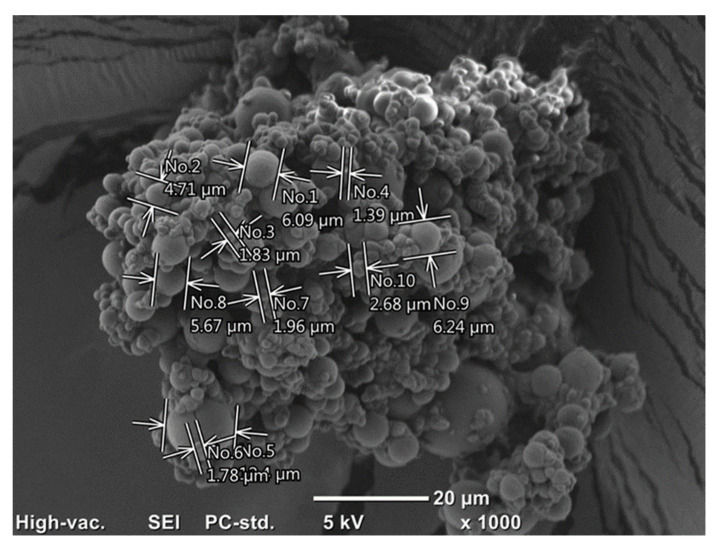
Microstructure of honeydew honey encapsulated using heteropolysaccharides isolated from rye bran HH-HPS as a coating material.

**Figure 12:**
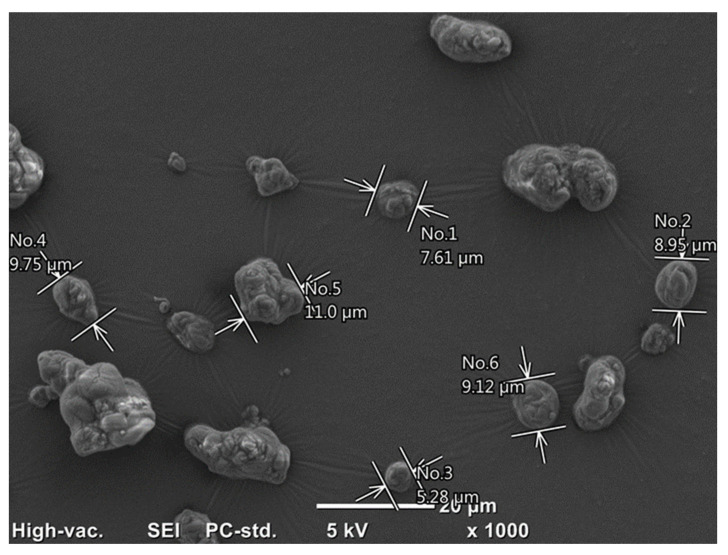
Microstructure of honeydew honey encapsulated using heteropolysaccharides isolated from rye bran RJ-HPS as a coating material.

Micrographic analysis of the tested bee-product encapsulates showed their varied morphological structure, as shown in Figure 11 and Figure 12, depending on the type of core material. Based on the microscopic analyses, the presence of particles with the smallest diameter was demonstrated in the encapsulates of preserved HH-HPS honeydew honey (average 2.3 µm). The largest microcapsule sizes were found for RJ-HPS (average 8.6 µm).

The tested encapsulates of bee products showed differences in the surface structure of individual particles. Desirable morphological properties of microcapsules include a uniform size and shape, which ensure consistent release rates and predictable behavior in applications, and a smooth surface texture is preferred to reduce aggregation and enhance stability. Additionally, a robust and flexible shell is essential to protect the core material from environmental factors and mechanical stress. These properties are crucial for the effective delivery and performance of the encapsulated substances in various industrial and pharmaceutical applications [28]. A particularly desirable morphology of microcapsules was obtained by encapsulating honeydew honey using heteropolysaccharide biopolymers from rye bran (HH-HPS, Figure 11). The encapsulates were characterized by optimal microcapsule morphology, i.e., a uniform, spherical shape and a smooth surface. Nonetheless, the aggregation of microcapsule particles can significantly affect their performance as prebiotics. Aggregated particles may lead to uneven release and distribution of the prebiotic substances within the gastrointestinal tract, which can reduce their efficacy. This non-uniform release can impair the ability of prebiotics to selectively stimulate the growth and activity of beneficial gut microbiota. Additionally, aggregated particles may have altered dissolution and absorption profiles, potentially impacting the stability and bioavailability of the encapsulated prebiotics, thereby diminishing their health benefits [22]. The observed surface roughness of the RJ-HPS preparations may be the result of the crystallization of monosaccharides, i.e., fructose and glucose on the encapsulate’s surface, which takes place during the spray-drying process. Regardless of the type of core material subjected to encapsulation and the coating material used, the obtained SEM microscopic images for the analyzed fixed bee preparations indicated the presence of microcapsule particles in agglomerate-like formations, thus indicating a tendency for the encapsulated particles to be mutually adhesive.

He et al. [29] characterized the morphology of microcapsules of the fat fraction of royal jelly. The microcapsules obtained by the authors were characterized by a smooth surface, spherical, uniform shape, and particle diameter ranging from 10 to 30 µm. Suhag and Nanda [30] compared the influence of the type of carrier on the morphology of honey powders prepared with maltodextrin, gum arabic, or whey protein isolate, respectively. Microscopic analyses performed by the authors showed a high diversity in the obtained structures. The honey preparation containing maltodextrin was the only one characterized by forms with a porous surface and spherical shape, ranging in size from a dozen to several dozen micrometers. Honey powder particles obtained with gum arabic or whey protein isolate were characterized by irregular shape, surface roughness, and highly agglomerated forms [28]. Samborska et al. [31] carried out the process of low-temperature spray drying of rapeseed honey using maltodextrin (in the amounts of 20, 30, and 40%), respectively, with nutriose wheat fiber preparation (20%) and a mixture of both carriers (20%). The authors noted that the use of 30 and 40% of the carrier material, i.e., maltodextrin, in the spray-drying process of rapeseed honey resulted in obtaining a fixed preparation with the desired particle morphology, characterized by a uniform, spherical shape. Rapeseed honey powders obtained with a lower share of carrier material were characterized by highly agglomerated structures, the particles of which were connected with each other by numerous liquid bridges (so-called liquid bridges).

SEM analysis of the morphology of particles of the obtained bee-product encapsulates showed diverse morphological structures for the tested fixed bee-product preparations with a similar microstructure tendency to that indicated by Samborska et al. [28]—especially where the authors point to the appearance of agglomerated clusters of encapsulate particles, the preparation of which required a relatively small amount of carrier in relation to the amount of core. It should be mentioned here—which is also a great achievement of the conducted research—that the share of the heteropolysaccharide carrier in the discussed case was only from 2.7% to 20%, where this amount is usually from 40 to 60%. Regardless of the core material and the heteropolysaccharide biopolymer used in the encapsulation process, the obtained fixed preparations of bee products were characterized by particles with diameters of several micrometers; moreover, based on the obtained microscopic photos of all the tested microcapsules of bee products, the occurrence of mutual adhesion of the encapsulated particles, resulting in agglomeration of preparation particles, was noted.

## 3. Materials and Methods

### 3.1. Materials

Heteropolysaccharides (HPS), i.e., the water-extractable arabinoxylans fraction, were isolated from rye bran, a by-product of the milling industry. The extraction process of HPS was carried out according to a previously described method [16]. The centrifuged supernatant was reduced to half its volume on an evaporator (Heidolph Basis Hei-VAP ML, Hamburg, Germany) and subjected to 48-hour dialysis (dialysis tubes ≥ 12 kDa). The obtained retentate solutions were lyophilized using a freeze dryer type CHRIST Beta2-8 LSCplus, Berlin, Germany. Honeydew honey and royal jelly were sourced from a local apiary (Łysoń, Poland). 

### 3.2. Preparation of Honey- and Royal Jelly Loaded Microcapsules

The encapsulation process was carried out according to our own method as described in a previous study [32,33]. Based on these studies, it was shown that the optimal ratio of carrier in encapsulates is 4–25% for the core of royal jelly and honey. A carrier solution was prepared by dissolving 2.0 g HPS in distilled water so that the resulting solution had a concentration of 5% dry basis. The solution was stirred for 1 h at 20 °C. Next, 8 g of honeydew honey or 50 g of royal jelly were added to HPS solution. The mixtures were stirred on a magnetic stirrer for 22 h at 20 °C in order to cross-link the arabinoxylans fraction. The obtained mixtures were spray-dried to produce microcapsules of bee products, i.e., honeydew honey or royal jelly. The drying process was carried out using a Büchi Mini Spray Dryer B-290 (Hamburg, Germany) under the following conditions: temperature at the inlet 110 °C; temperature at the outlet 68 °C; flow velocity 3 mL min^−1^. Each time, three independent drying processes were performed.

### 3.3. Characterization of Microcapsules

#### 3.3.1. Analysis of the Prebiotic and Pro-Adhesive Properties of Bee Products Encapsulates

##### Biological Material

The research material used in this work consisted of 5 strains of lactic acid bacteria (LAB): *Lactiplantibacillus plantarum* ŁOCK 0981, *Levilactobacillus brevis* ŁOCK 0983, *Lactobacillus delbrueckii* ŁOCK 0987, *Lacticaseibacillus paracasei* ŁOCK 0993, and *Lacticaseibacillus rhamnosus* ŁOCK 0908 from the collection of the Institute of Fermentation Technology and Microbiology (LOCK 105), Lodz University of Technology, Poland. One strain of LAB (*Loigolactobacillus coryniformis* 10AN) and nine strains from the genus *Bifidobacterium: Bifidobacterium* BB-12 from CHR Hansen, *Bifidobacterium animalis, Bifidobacterium longum, Bifidobacterium infantis*, *Bifidobacterium* sp. *6512, Bifidobacterium* sp. *01/01/76*, *Bifidobacterium* sp. *02/01/73*, *Bifidobacterium bifidum*, and *Bifidobacterium breve* derived from the collection of the Department of Environmental Biotechnology, Lodz University of Technology.

##### Strain Activation and Storage

To activate the biological material used in the assays (points 2.3.1.1., 2.3.1.2., and 2.3.1.3.) the bacterial strains were thawed and transferred to the fresh liquid media: DeMan, Rogosa, Sharpe broth (MRS forlactic acid bacteria), and Bifidobacterium medium (for *Bifidobacterium* strains). Then, depending on the origin of a given strain, the samples were incubated for 48 h at 30 °C or 37 °C. Bacteria were passaged 3 times using a 3% inoculum for this purpose and then cultured for 48 h at 30 ° C or 37 °C. Bacteria were used for the analysis from the third or fourth passage.

For bacterial storage, a 24-hour culture of the probiotic strain in MRS or *Bifidobacterium* medium was centrifuged (3468 g, 10 min, 22 °C). The supernatant was decanted, the resulting pellet was washed with sterile PBS buffer, then resuspended in a suitable medium with 20% glycerol. The obtained biomass was poured into sterile Eppendorf tubes. The strains were stored at −20 °C.

##### Influence of Native Honeydew Honey, Royal Jelly, and Their Microcapsules on Growth of Lactic Acid Bacteria and Bifidobacterium Strains

In order to define the influence of the innovative encapsulation of honey and royal jelly on the growth and decline phase of selected LAB and *Bifidobacterium* spp. strains, an inoculum was introduced into the modified glucose-free MRS medium or Bifidobacterium medium, which consisted of 48-hour bacterial cultures and a 3% extract of bee products or their encapsulates at a concentration of 0.01 g mL^−1^. The control sample for each strain was a culture of bacteria in unmodified medium. Depending on the origin of the tested strain, the samples were incubated at 30 °C or 37 °C. Samples were placed in physiological saline solutions starting at zero hour (initial time) and successively on days 2, 7, 14, and 21 of incubation. Bacterial density was determined by the Koch plate method from successive 100-fold dilutions of selected strains. Petri dishes were incubated at temperatures optimal for the tested bacterial strains (30 °C or 37 °C) for 72 h, and then the grown colonies were counted. The result is given as the logarithm of the colony forming units N [CFU mL-1]—logN. The test was performed in three independent replications.

##### Adherence of Bacteria to Abiotic and Biotic Surfaces—Biofilm Formation

The ability of the LAB and *Bifidobacterium* spp. to form biofilm on abiotic (polystyrene) and biotic (collagen and porcine mucous) surfaces was also determined, according to the methods published by Koryszewska-Bagińska et al. [34] with slight modification [34,35].

Suspension of each active strain in PBS was centrifuged at 3468× *g* RCF for 10 min and washed, then, the absorbance (A) was adjusted to 1.0 at a wavelength of 630 nm. The binding assay for polystyrene was performed on unmodified 96-well polystyrene microplates (Corning Inc., Mszczonów, Poland), whereas adhesion to collagen was analyzed with commercially available 96-well polystyrene microplates with a collagen coating (Corning^®^ BioCoat™ Collagen I; Corning Inc.). To determine the strains adhesion ability to porcine mucous, 6-well microplates (Corning Inc.) were coated (60 min, 37 °C and 24 h, 4 °C) with 150 mg/mL of mucous from porcine stomach solution in PBS (Type II; Sigma-Aldrich, St. Louis, MI, USA; 72 h, 4 °C), respectively. The excess of unbounded mucous was removed and wells were washed with PBS. Bacterial suspensions were added to the microplates in 3 (6-well plates) or 8 (96-well plates) repetitions. The plates were incubated for 2 h at 37 °C, and unattached cells were washed with PBS. Next, adhered cells were fixed with 80% (*v*/*v*) methanol (15 min for polystyrene and collagen adherence assays) or at 60 °C (20 min for porcine mucus adherence assays) and stained with 0.1% (*w*/*v*) crystal violet for 15 min. Subsequently, wells were rinsed with PBS and then the pigment was washed out from attached cells on an orbital shaker (Chemland, Stargard Szczeciński, Poland). For pigment removal, 96% (*v*/*v*) ethanol (for polystyrene and collagen adherence assays) or 20 mM of citrate buffer (for porcine mucus adherence assays) was used. Absorbance was measured with a TriStar2 S LB 942 microplate reader (Berthold Technologies GmbH and Co. KG, Hamburg, Germany) at wavelengths of 490 nm (for polystyrene and collagen adherence assays) or 570 nm (for porcine mucus adherence assays). The adhesion ratio was calculated based on the equation:

$$Adhesion\;ratio=\frac{A_{Sample}}{A_{Control}}$$

where

*A_Sample_* is the absorbance of the sample; and *A_Control_* is the control sample’s absorbance (PBS solution added to wells).

##### Short-Chain Fatty Acid (SCFA) Analysis

High-performance liquid chromatography (HPLC) analysis was carried out using an HPLC+ Ultimata 3000 Dionex equipped with a UV-Vis detector and an A11606 C18 column (2.1 × 7150 mm) (ATC, USA). The flow rate was 0.8 mL/min, the temperature was 30 °C. Phosphorus buffer (pH 2.34) was prepared by dissolving 2.4 g of NaH2PO4 in 1 L of water, to which 1.4 mL of 85% phosphoric acid (phase A) and acetonitrile (phase B) were added. The following gradient program was used: 0 min A:100%, 10.5 min A:20%, 19.5 min A:100%. Identification and quantification of compounds were carried out based on standard compounds: acetic acid, propionic acid, and butyric acid (Sigma) [36].

#### 3.3.2. Surface Morphology Assessment with Scanning Electron Microscopy (SEM)

The surface morphology and microstructure of the obtained microcapsules were studied by scanning electron microscopy (SEM). SEM images were recorded using a Jeol-JCM-6000 scanning electron microscope (Japan). The examined samples were sputter-coated with gold under a vacuum (without any noble gas). Images were recorded at differences of acceleration potentials ranging from 5 kV to 10 kV mode [18].

### 3.4. Statistical Analysis

Unless stated otherwise, all the biological results are presented as means of 3–12 repeated experiments ± SD. All calculations were evaluated for significance using one or two-way ANOVA followed by Dunnett’s test with the GraphPad Prism 6.0 software (GraphPad Software Inc., La Jolla, CA, USA). *p* ≤ 0.05 was considered statistically significant.

## 4. Conclusions

The use of innovative carriers, i.e., natural bioactive biopolymers isolated from rye bran, made it possible to obtain preserved bee products of honey and royal jelly in the form of encapsulates, which at the same time ensured an increase in the biostability of the bioactive components of the microcapsule core material and their protection against the adverse effects of external environmental factors. Moreover, the preparation of fixed bee products using innovative heteropolysaccharides allowed for a significant reduction in the share of the carrier in the encapsulates, in relation to the commonly used amount of coating material in the process of spray drying honey and royal jelly. This type of heteropolysaccharide has not been used so far as a carrier in the encapsulation process. These biopolymers enable to significantly reduce the content of the carrier in the microcapsules.

Characterization of the microstructure of bee product encapsulates, determined using the SEM scanning electron microscopy technique, showed that the obtained microcapsules were characterized by spherical, regularly shaped particles with a smooth surface and diameters of several micrometers.

The obtained microcapsules of bee products, i.e., honeydew honey and royal jelly, showed potential prebiotic and pro-adhesive properties towards bacteria of the genus *Bifidobacterium* spp. and lactic acid bacteria. Additionally, as a result of fermentation of fixed preparations of bee products in the lumen of the large intestine, an increase of 39% in the synthesis of microbial metabolites in the form of SCFAs, including butyric acid, was observed compared to the amount of SCFAs obtained in the fermentation of native bee products. Butyric acid is of great importance as it may stimulate the secretion of mucus with a protective effect on the digestive tract and trigger the growth and differentiation of intestinal epithelial cells.

Summarizing the results of the analyses presented in this work, it was found that obtaining microencapsulated preparations of honey and royal jelly is possible and allows for maintaining the features of the mentioned bee products to the greatest extent possible, and even increasing their bioactivity as a result of the use of innovative heteropolysaccharide carriers isolated from rye bran. In order to achieve the aim of the work, innovative preparations of honey and royal jelly in the form of microcapsules were obtained with a wide range of potential applications. Possible directions of use include a preparation supporting wound healing or a nutraceutical with immunomodulatory and prebiotic properties, with the possibility of controlled release in the matrix.

## Data Availability

Data is contained within the article.
